# Surgical Management of Intracanal Rib Head Dislocation in Neurofibromatosis Type 1 Dystrophic Kyphoscoliosis: Report of Two Cases and Literature Review

**DOI:** 10.1155/2016/2908915

**Published:** 2016-06-30

**Authors:** George I. Mataliotakis, Nikolaos Bounakis, Enrique Garrido-Stratenwerth

**Affiliations:** Royal Hospital for Sick Children, Scottish National Spine Deformity Centre, Sciennes Road, Edinburgh EH9 1LF, UK

## Abstract

There is still no consensus on the management of severe intracanal RH dislocation in neurofibromatosis type 1 dystrophic kyphoscoliosis. This study notes the early cord function impairment signs, reports a serious complication in a susceptible cord, identifies possible mechanisms of injury, and discusses the management of intracanal RH dislocation presented in the literature. First report is as follows: a 12-year-old female with cord compromise and preoperative neurology that underwent thoracotomy and anterior release. The RH was left in situ following a rib excision. During the posterior stage of the procedure she presented with complete loss of all IOM traces prior to any correction manoeuvres. The neurology recovered 72 h postop and the final correction and instrumented fusion were uneventfully completed 15 days postop. Second report is as follows: a 10-year-old male, whose only neurology was a provoked shock-like sensation to the lower limbs following direct pressure on the rib cage. He underwent an uneventful posterior RH excision and instrumented correction and posterior spinal fusion. In conclusion, any possible cord dysfunction sign should be sought during examination. Decompression of the spinal cord by resecting the impinging bony part, even in the absence of neurological symptoms, is advised before any attempt to release or correct the deformity.

## 1. Introduction

Neurofibromatosis type 1 (NF1), also known as von Recklinghausen's disease, is a single gene hamartomatous disease inherited by the autosomal dominant trait [1, 2]. The relentless deterioration of the short dystrophic curves, which leads to acute kyphosis and possible vertebral subluxation, mandates surgical stabilization [1, 3]. Intracanal rib head (RH) dislocation at the convex of the dystrophic curve may impinge on the cord and constitute another cause of neurology [3–9]. The “painful rib hump” sign caused by the RH dislocation has recently been described [8].

Even though there are recent reports of retraction of the RH away from the cord along with curve correction [2, 10, 11], there is still no consensus among authors that the excision of the dislocated RHs is indicated in symptomatic NF1-dystrophic curves [5, 8, 12, 13]. The preoperative identification of a “cord at risk,” the susceptibility of the cord to intraoperative manipulations, and the sequence of an iatrogenic injury are not fully described yet.

We report two selected cases: a case of intraoperative neurological injury in a previously symptomatic patient with cord changes and a case of “hidden” neurology in an otherwise asymptomatic patient. The purpose of this study is to report a serious complication, identify a possible mechanism of injury, highlight the importance of early neurology, and discuss management of intraspinal RH dislocation.

## 2. Case  1

**Figure 1:**
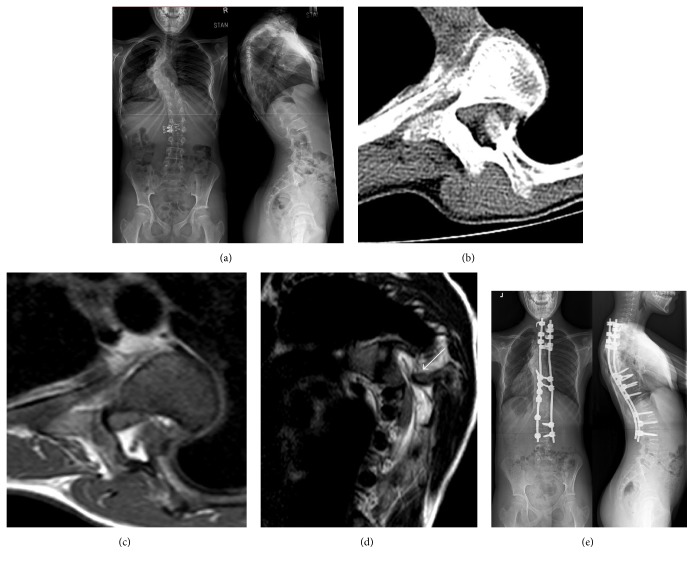
Preoperative whole spine AP and lateral X-ray (a). Preoperative CT (b) and axial T2 MRI (c) demonstrating the right 6th rib head intracanal dislocation and cord impingement. (d) Preoperative sagittal T2 MRI views demonstrating the rib head cord impingement and the flattening adjacent to the acute kyphosis (white arrow). Whole spine AP and lateral (e) X-rays 2 years postoperatively.

A 12-year-old premenarchal girl presented with back pain due to dystrophic NF1 left thoracic kyphoscoliosis (Figure 1(a)). She had bilateral brisk patellar reflexes and a left four beat ankle clonus. The patient reported shooting pain into her legs on deep forward bending. Preoperative MRI and CT scans revealed a flattened cord at the apex of the kyphosis with a penetrating left 6th RH adjacent to the cord without compression (Figures 1(b)–1(d)).

A combined fusion and posterior RH excision were planned with the aid of multimodal spinal cord monitoring. Following a thoracotomy the left 6th rib was excised, leaving the neck and head in situ. During the T4–T9 discectomies a transient loss of Motor Evoked Potentials (MEPs) occurred bilaterally in lower limbs, which responded to an increase in the mean arterial blood pressure. Somatosensory Evoked Potentials (SSEPs) remained normal.

Normal reference traces were present at the beginning of the second stage: posterior instrumented spinal fusion. After completion of the instrumentation and prior to correction manoeuvres all MEPs and SSEPs were lost completely. Following a laminectomy, the dislocated RH, which was not adherent to the dura but was impinging on the cord, was excised. Wake-up test showed no spontaneous movement in the lower limbs with good upper limbs movement.

Postoperative neurological examination showed grade 3 muscle power (MRC grading) in all muscle groups of the left lower limb. Right lower limb was normal. Fine touch and proprioception remained intact bilaterally. 48 h postop MRI scan showed no evidence of cord signal changes. 72 h postop the patient regained normal muscle power and urinary continence.

Fifteen days postoperatively the patient underwent posterior correction of the deformity. The MEPs remained stable during the procedure. Postoperative radiographs evidenced a main thoracic curve of 45° (51% correction). Lateral radiographs and CT scan confirmed a correction of the thoracic kyphosis to 48° (36% correction). At 2-year follow-up (Figure 1(e)) there has been no significant loss of correction and the patient remains asymptomatic.

## 3. Case  2

**Figure 2:**
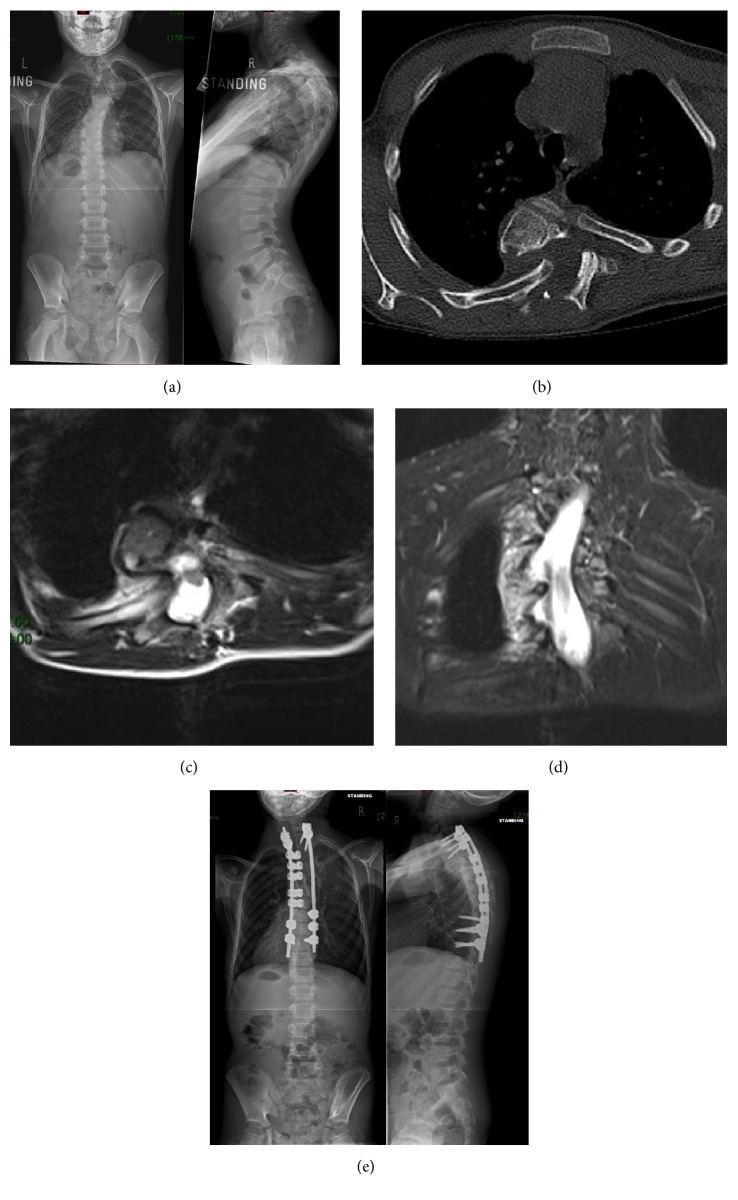
Preoperative whole spine AP and lateral X-ray (a). Preoperative CT scan (b) demonstrating the rib head intracanal dislocation. Preoperative sagittal T2 MRI axial (c) and coronal (d) views demonstrating the rib head in close proximity to the cord but without impingement. Postoperative whole spine AP and lateral X-rays (e).

A 10-year-old boy presented with a rapidly progressive spinal deformity and scapulae asymmetry, due to dystrophic NF1 sharp angular proximal thoracic kyphoscoliosis (Figure 2(a)). There was no neurologic deficit. Interestingly, the patient complained of discomfort only when lying on his right side with shock-like sensations to his lower limbs bilaterally. The CT/MRI imaging shows intracanal dislocation of the right 4, 5, and 6th ribs, with the 5th being in contact with the spinal cord and no cord-substance high signal present (Figures 2(b)–2(d)).

He underwent posterior resection of the 5th RH through a hemilaminectomy approach. Following alar/coronal ligament release, the RH was extracted with stable spinal cord monitoring traces. The posterior instrumented spinal fusion was then completed uneventfully (Figure 2(e)). A CTLSO was used postoperatively.

## 4. Discussion

The canal expansion due to dural ectasia is probably protective, regarding the early development of neurological symptoms [1]. Apart from possible neurofibromas, the acute kyphotic deformity and instability may lead to neurology compromise due to secondary cord injury [1–7, 9, 12, 14, 15].

The dislocation of the RH into the canal is caused by the insufficiency of the costovertebral/costotransverse articulation and medial-ward pressure by the thoracic cage [1–3]. It takes place through the enlarged neural foramina at the apex of the convexity of the curve [2]. The RH may impinge on the cord [3–9] and may cause neurology [3, 7–9]. Gkiokas et al. [8] described clearly the mobile RH, which causes neurology and pain in an otherwise neurologically intact patient, introducing the “painful rib hump” sign. Lhermitte's type phenomenon might represent early sign of cord irritation, manifesting as shock-like sensation to lower limbs [4, 12]. This was the only clinical finding on our 2nd case, indicating an unstable RH and possible early cord injury.

MRI with contrast is the imaging modality of choice for soft tissue lesions in NF and the T2W sequences may demonstrate the intraspinal RH dislocation [4]. However, there are reports where preoperative MRI failed to diagnose the intraspinal RH dislocation leading to postoperative neurological injury [5, 9, 15]. The CT scan can reliably demonstrate the intracanal RH dislocation because of better delineation of bony anatomy [3, 13]. The preoperative evaluation of the imaging should be cautious, as the RH and spine positions without gravitational forces may underestimate the degree of canal intrusion.

Surgical treatment is difficult due to excessive bleeding and the distorted anatomy. High pseudoarthrosis rates have been reported with posterior-only fusion in dystrophic scoliosis and therefore generous anteroposterior fusion has been advocated for curves exceeding 50° of kyphosis [16].

No consensus exists in the literature regarding the surgical sequence for the treatment of intraspinal RH dislocation and scoliosis correction.

A literature review reveals 16 publications with a total of 49 patients with NF1 and intraspinal dislocation of the rib (Table 1). Most of the patients were teenagers with a mean age of 13 years (average: 13.45 ± 5.72 years, range: 6–41 y) with almost 1 : 1 male to female ratio (25f : 23m). Neurological status was reported in 24 cases. Neurological deficits were present in 50% of the reported cases. Two or more ribs penetrating into the canal were presented into 43% of the nonsymptomatic patients and in 42% of the symptomatic patients. In 47% (9/19) of the reported cases the RH was in close proximity or was impinging on the spinal cord. Six of the nine patients with a RH in close proximity to the spinal cord underwent resection. Of the three patients in whom the RH was not removed during scoliosis fusion, two patients developed delayed neurological deficits requiring subsequent decompression. The intraoperative monitoring traces were lost in the patient who had his RH left in situ and the distal rib resected [7].

Among all the reported cases with intracanal dislocation, 32 patients received PSF only; one had noninstrumented in situ PSF [5] and one had instrumented in situ PSF following previous correction by halo traction [6]. The in situ noninstrumented PSF led to lower limb weakness and paraparesis 6 weeks postoperatively with the authors not clarifying the cause of the neurology deterioration [5]. There were 19 staged operations with 11 of them being combined anterior and posterior. Of the 11 patients, 8 had ASF and 6 underwent the anterior procedure first.

RH excision is generally advised routinely in most of the case reports [3, 7, 14]. The posterior approach offers better visualisation than the unilateral exposure through an anterior approach [13].

Mao et al. [10] and Sun et al. [11] showed that spontaneous RH reduction occurred, following curve correction. Yalcin et al. [2] also observed under direct vision how the RH migrated out of the canal during scoliosis correction. They concluded that, in the presence of neurological symptoms or evidence of compression, resection of the rib prior to any surgical manipulation (release or correction) is necessary [2]. In asymptomatic patients with no evidence of spinal cord compression, RH excision was considered questionable. Yalcin et al. [2] suggested direct visualisation of the RH via hemilaminectomy during correction manoeuvres.

Abdulian et al. [6] offer a different point of view, advocating resection of every intracanal RH dislocation. In addition, the authors recommended rib shaft osteotomy in cases where the RH remains unresectable because of cord adhesions.

Similar to our case 1, in the report by Mukthar et al. [7] the spinal cord monitoring traces were lost following resection of the rib shaft and leaving the RH in situ. According to Leung et al. [17] a spinal cord at risk is more likely to demonstrate intraoperative monitoring changes and those changes are twice likely to be associated with postoperative neurological deficit. Cheh et al. [18] reported loss of MEPs in 21% of paediatric kyphosis correction which was attributed mainly to hypotension, overcorrection, or combination of the two and was completely reversed by increasing the mean arterial pressure or reducing the magnitude of correction. Shimizu et al. [19] in their recent animal study showed that severe kyphosis causes demyelination, reduced blood supply, and neuronal loss of anterior horn cells.

Several factors may have contributed to the transient neurological injury to herein reported case 1. Vascular insufficiency during the anterior discectomy stage may have caused the drop of the IOM traces, as they recovered after an increase in the mean arterial blood pressure. The RH was not removed during the anterior stage as this would result into greater haemorrhage and possible further secondary vascular insult to the cord. Also, there is still no strong evidence in the literature to suggest a direct injury to the cord by a remaining RH stump. The subsequent prone position and the posterior facetectomies may have contributed to a degree of spinal instability and alteration of the kyphoscoliotic angle. Also, the change in alignment with translation of the spinal cord towards the convexity may have produced compression by the penetrating RH. Furthermore, the RH, having lost its lateral stabilizers due to the thoracotomy and rib resection, might have adopted a new medial position further impinging on the cord. Similar to our second case, it may be safer to relieve the cord from the RH compression, prior to any release or correction, which will change the relationships in the acute kyphos.

We postulate that the flail RH, left in the spinal canal following a rib-only resection, may be risky, because it loses the stability provided by the rib cage and because it may potentially change position. Secondly the loose RH will not reduce by ligamentotaxis during translational correction manoeuvres. In cases where the RH remains unresectable, because of cord adherence, translational correction manoeuvres should probably be avoided. We also believe that even asymptomatic cases without gross MRI cord signal changes should still be investigated for subtle signs of cord impairment, which might render it vulnerable during correction manoeuvres.

Even though the intracanal RH dislocation is a well-documented manifestation of the NF kyphoscoliosis, its severe form is not frequent enough for any conclusion to be supported by large number of cases. However, the conclusion is reasonable and is in line with the experience presented in all reports on the same topic in the literature. Preoperatively, we would suggest a thorough clinical investigation for cord impairment signs (“painful rib hump,” Lhermitte's-like, etc.) and precise imaging for the intracanal RH position in relation to the cord. We would recommend excision of the RH, if in close proximity to the spinal cord, prior to attempting anterior spinal release or posterior correction manoeuvres. This sequence will also aid in the correct identification of the cause of a possible intraoperative IOM event.

## 5. Conclusion

Rib head intracanal dislocation is a dystrophic feature of patients with NF1 scoliotic curves. The protruding part of the rib although usually asymptomatic may cause neurological impairment by impinging on the spinal cord.

Provoked neurological signs should be sought during clinical examination in order to identify any cord dysfunction. CT and MRI scans should be performed to diagnose the extent of rib head penetration or cord involvement and to assist in surgical planning.

Decompression of the spinal cord by resecting the impinging bony part, even in the absence of neurological symptoms, is advised before any attempt to release or correct the deformity. This strategy seems to be the safest and will aid the surgeon and the neurophysiologist in discriminating the cause of possible positive IOM events during surgery.

## Figures and Tables

**Table 1:** Surgical management of intracanal rib head dislocation in neurofibromatosis type 1 dystrophic kyphoscoliosis.

Author	Age (range)/sex	Dislocated ribs [*N* of ribs (*N* of patients)]	Cord impingement	Other lesions present	Preoperative neurology	Operation details	Rib heads resection	Complications after 1st operation	Neurology recovery
Flood et al. 1986 [12]	13	>2	No	Yes	Knee and ankle clonus	Two-stage vertebral wedge resection with rib excision and fusion. Traction used perioperatively, PSF	Yes	NR	Residual clonus

Major and Huizenga 1988 [13]	13f	2	No	No	Transient loss of sensation below the waist and inability to move LL after fall on rib hump	Two-stage ASF with RH resection followed by segmental instrumented PSF	Yes	NR	n/a
	5f	2	No	No	No	Anterior interbody fusion with RH resection followed by segmental instrumented PSF	Yes	NR	n/a
	11m	1	No	No	No	Posterior fusion with RH resection	Yes	NR	n/a

Deguchi et al. 1995 [9]	12f	2	Yes	No	Weakness of the LL, difficulty walking with eventual paraparesis, hypesthesia below waist, ankle clonus, and knee/ankle HR; gradual	Laminectomy and proximal resection of the compression rib; two-stage combined ASF and instrumented PSF; dislocated RH was resected	Yes	NR	Yes

Dacher et al. 1995 [15]	10f	1	No	No	Bilateral ankle clonus and daytime micturition	Two-stage SF with CD instrumentation	NR	NR	Yes

Kamath et al. 1995 [20]	13m	1	No	Yes	No	Intraspinal RH resection with right T9/10 hemilaminectomy and instrumented PSF	Yes	NR	n/a

Khoshhal and Ellis 2000 [5]	16m	1	Yes	Yes	No	In situ noninstrumented PSF; revision: anterior decompression and RH resection 8 months postop due to residual neurology	No	Progressive LL weakness, spasticity, and being unable to walk	Residual HR

Legrand et al. 2003 [21]	13m	1	NR	NR	Hyperreflexia	PSF & ASF	No	NR	NR
	10f	2	NR	NR	No	NR	Yes	NR	n/a
	16m	1	NR	NR	Hypotonia	PSF & ASF	No	NR	Yes
	41f	2	NR	NR	Pyramidal tract syndrome	Halo traction and RH resection	Yes	NR	Yes

Mukhtar et al. 2005 [7]	10m	1	Yes	No	Back pain induced by movements; weakness and shock-like feeling in Rt LL on direct pressure of Rt side of torso; gradual	Posterior partial rib resection with RH left in situ; 2nd op: posterior in situ fusion (T6–T11)	No	Due to IOM changes the RH was left in situ and the rest of the Rib was excised	Yes

Gkiokas et al. 2006 [8]	13f	1	Yes	No	B/L Babinski, clonus, weakness in LL (foot drop), decreased sensation, HR, and daytime micturition; “painful rib hump” symptoms	Posterior decompression and resection of the RH, PSF	Yes	No	Yes

Yalcin et al. 2008 [2]	14m	2	No	Yes	No	Posterior laminectomy and PSF	Yes	No	n/a
	12f	2	Contact	Yes	No	Posterior laminectomy and PSF	No	No	n/a
	6m	2	No	NR	No	Anterior 5 level annulotomy and resection of T10 and T11 ribs; RH left in situ; growing rod construct	No	No	n/a

Cappella et al. 2008 [3]	14m	1	Yes	NR	Gradual weakness in lower limbs	Staged posterior instrumented and anterior SF with casting; revision: posterior decompression	No	Progression of deformity	Yes

Ton et al. 2010 [4]	14m	2	No	Yes	Back pain, knee and ankle HR, and clonus and “painful rib hump” like symptoms	T4 laminectomy and posterior fusion and instrumentation	Yes	NR	NR
	11f	1	Yes	No	No	Multilevel discectomies, T9 laminectomy, RH resection, and PSF	Yes	NR	n/a
	11m	1	No	No	No	T9 laminectomy, ASF, and PSF and 9th RH resection	Yes	NR	n/a
	9f	1	Yes	Yes	Back pain, R foot weakness, and B/L LL HR and clonus	Resection of neurofibroma and 6th RH, PSF, & ASF	Yes	NR	NR

Abdulian et al. 2011 [6]	14m	2	Yes	No	No	1st op: posterior T5 hemilaminectomy and T5/6 facetectomy, 2nd op: posterior T6 hemilaminectomy and T6/7 facetectomy, 3rd op: anterior T4–T9 release, and 4th op: T2-L3 instrumented PSF	Yes	The 2nd op was because the next intracanal protruding rib was missed	n/a

Krishnakumar and Renjitkumar 2012 [22]	11f	2	NR	NR	NR	PSF	Yes	NR	NR

Sun et al. 2013 [11]	13, 4f/2m	NR	NR	NR	No	SPOs and posterior correction with PSF	No	No	n/a

Mao et al. 2015 [10]^*∗*^	13 (8–33), 10f : 9m	1 (12), 2 (6), 3 (1)	NR	NR	No	The posterior correction could be alone or adjunct with perioperative traction and occasionally supplemented with SPO; the anterior stage could include anterior release or convex growth arrest or ASF. 13 posterior only and 6 anterior & posterior	No	NR	n/a

This table shows all published studies in the English literature to date, which are reporting on the management of intracanal rib head dislocation in neurofibromatosis type 1 dystrophic curves; level of evidence (LoE) V, ^*∗*^case series: (LoE) IV, PSF: posterior spinal fusion, RH: rib heads, and LL: lower limbs. Op: operation.
